# A Hybrid FMCDM Approach for the Evaluation and Selection of Homestays

**DOI:** 10.3390/ijerph19148688

**Published:** 2022-07-17

**Authors:** Tsung-Yu Chou

**Affiliations:** Department of Distribution Management, National Chin-Yi University of Technology, Taichung 41170, Taiwan; arthur@ncut.edu.tw

**Keywords:** tourism, homestay, fuzzy multiple-criteria decision making, means-end chain, fuzzy decision making and trial evaluation laboratory, fuzzy analytic hierarchy process

## Abstract

Due to the beautiful rural scenery, rural tourism has gradually become a popular trend as affected by urbanization in Taiwan. The purpose of this study is to develop an objective and systematic evaluation model for homestay selection in Taiwan. Specifically, this study combines three fuzzy multiple-criteria decision-making (FMCDM) methods of means-end chain (MEC), fuzzy decision making and trial evaluation laboratory (FDEMATEL), and fuzzy analytic hierarchy process (FAHP). First, we use MEC to obtain important factors for homestay travelers to choose homestays, then we extract key influencing factors by FDEMATEL method, and finally, we rank these factors in importance by FAHP to provide travelers and managers with a reference. In addition, this study combines fuzzy theory to avoid the bias caused by human subjective judgment, so as to solve the problem of objectivity in the selection and evaluation model of homestay criteria. According to the results of our case studies, a clean environment, professional service staff, safe facilities and beautiful buildings are the key factors for consumers to choose homestays. The results of this study can provide a reference for homestay managers to understand the priorities of travelers and optimize customer service in Taiwan.

## 1. Introduction

The tourism industry is viewed as a “smokeless industry” since its output value is based on tourist visits rather than the establishment or construction of a factory. For developing countries, tourism has long been seen as a path to sustainable development [1]. This will then promote the national economy and create employment opportunities. Since the Taiwanese emphasize life and leisure now, faced with beautiful rural scenery in Taiwan, rural tourism has gradually become a popular trend as affected by urbanization. Homestay tourism can not only meet the personalized accommodation needs of guests, but also play a key role in promoting economic growth and protecting traditional culture and arts [2,3]. In recent years, Taiwan’s government has vigorously promoted the advance of leisure agriculture to transform agriculture from perspectives in the farming, fishery, humanities, landscape, sightseeing, etc. Recently, the development of leisure agriculture has brought many tourists. To provide accommodation for rural sightseeing tourists, most localities have begun to build homestays as an alternative to inns and hotels. Therefore, the integration of home-based management and local characteristics has created an opportunity to transform the rural economy. According to tourism statistics, the ratio of domestic tourism for Taiwanese in 2020 was 88.4%, the average number of domestic trips was 6.74 trips per person, and the average number of stay period was 1.54 days. Due to the impact of COVID-19, compared with 2019, the ratio of domestic tourism decreased by 2.7%, and the number of domestic trips decreased by 1.25 trips per person, but the average length of stay increased by 0.03 days [4]. This explains why homestay practitioners devote resources to this industry. However, homestay owners do not know much about what tourists expect. Therefore, this study will be explored to understand the key factors of tourist demand, and it is important to give operators an important reference direction.

In 2011, Taiwan’s government incorporated the development of cultural and creative industries into the national development plan for expansion through scientific, technological, and cultural means. By employing both its intellectual and cultural capital, Taiwan added creative and aesthetic values into product development and daily life, providing many domestic industries, including the hotel industry, with self-transformation, growth, and business development opportunities. The cultural integration laid the foundation for hotels’ or homestays’ product design and service innovation, which showcased the local cultural values and characteristics, creating unique competitiveness for inns [5]. Therefore, inns can use unique characteristics such as local cultural characteristics, the natural environment, or artistic resources to attract customers’ attention and provide customers with pleasant artistic and cultural experiences [6,7]. Hence, for current characteristic homestays, in addition to personalized and life-oriented architectural design, its surrounding environment and services could improve the satisfaction and loyalty of consumers [8]. Although some studies have explored topics such as consumers’ decision making, consumer behavior intention, and selection attributes in choosing hotels [9,10,11], or investigated the customers’ perceived value of chosen hotels or the provision of selection decision-making models [12,13], only a few have explored the establishment of evaluation criteria for decision-making models combined with consumers’ perceived value. This is also the main motivation and purpose of this study to assist in establishing a decision-making model for homestay tourists in Taiwan.

MCDM provides an evaluation framework that can solve practical problems by helping decision makers to obtain effective solutions through scientific analysis methods. Combining the application of different tools to build a good evaluation model will be presented in this article. In the study, the means-end chain (MEC) method was used to obtain the decision-making attributes of tourists combined with consumers’ selection attributes and the perceived value of homestays. MEC is a way to examine consumer behavior and perceived value. It is typically used to investigate consumer consumption behaviors and the meanings that consumers attribute to products or services [14], and to establish a decision-making criteria framework based on the relationship between these meanings and cognitive value [15]. Therefore, this study used MEC to acquire decision-making attributes of consumers to establish a preliminary evaluation framework. In addition, the DEMATEL was employed to determine the relationship between dimensions or clusters and the core criteria for representative elements/dimensions. The criteria can be divided into visual cause–effect diagrams to highlight important criteria items that require urgent improvement. Therefore, under limited resources and time constraints, decision makers can focus on the factor group to obtain the most influential criteria and construct improvement strategies to yield twice the result with half the effort. In the research, this method is used to extract the important key factors from the criteria obtained by MEC. Finally, since the AHP method being the most widely used and well known [16,17,18]. In this study, AHP was used to establish a decision-making model from the key factors extracted by DEMATEL.

As consumers tend to form subjective ideas when making decisions, the related issues may involve a certain degree of fuzziness and uncertainty; that is, consumers are faced with the arduous task of evaluating and selecting various criteria and alternatives. The fuzzy set theory, one of the methods in assisting decision making [19], can effectively express the fuzziness in thinking and the preference of decision makers (DMs) [20]. Therefore, incorporating fuzzy theory into the method of this study will make the model more flexible and objective. Unlike previous studies based on the combination of DEMATEL and AHP, this study firstly used FDEMATE to locate important attributes that can affect consumer value. Then, FAHP was applied to carry out the weight estimation of each influential attribute because the selection of characteristic homestays is mostly subjective, and strong correlations can be found among attributes. Hence, the important decision-making criteria of homestays were determined, which can be utilized as a reference for homestay owners.

The major contributions and innovativeness of this paper are: first, based on the MEC theory, this paper obtained the attributes of consumers’ decision making on homestays through in-depth interviews with consumers, and it figured out the connections between the attributes and values to make them closer to consumers’ thoughts. This can function as a reference for consumers and homestay practitioners when deciding and selecting a characteristic homestay in the future. Second, MEC, FDEMATEL, and FAHP were integrated innovatively together for decision making. Significantly, the combination of fuzzy set theory made it feasible to obtain people’s real perception of fuzzy and subjective evaluation. Third, using the evaluation model to establish and rank key attributes/criteria for the consumer evaluation of characteristic homestays more accurately and objectively, this paper contributes to the business operation strategy of practitioners and provides a convenient method for the decision making for consumers.

This research aims to propose a hybrid FMCDM approach model for customers to help them decide when selecting a homestay. The structure of this research is as follows: Section 2 elaborates on the current status of Taiwan’s homestays and related literature on MCDM; Section 3 describes the research methods and the operation process of this paper; Section 4 explains the applications of the proposed method; Section 5 provides the theoretical and practical significance of the proposed method; finally, the conclusions and suggestions are put forward in Chapter 6 according to the obtained results.

## 2. Literature Review

### 2.1. Cultural and Homestays in Taiwan

Although there is no uniform definition of homestay, it is generally defined as a type of accommodation where tourists stay in the homes of local residents [21,22]. Homestay families rely on a good ecological environment and unique cultural customs, and use vacant houses to provide tourists with accommodation, so as to experience the local natural resources, lifestyle and cultural atmosphere [2,3]. According to Ahmad et al. [23], homestay tourism refers to a tourism model based on ecotourism and community tourism, where tourists will stay in homestays in rural areas, allowing them to participate in daily activities and learn and understand the local culture. Homestay has become an important research field for scholars in recent years. Relevant literature points out that scholars have discussed the behavioral characteristics of homestay operators [24], guest experience [22], homestay marketing [22,25] and the impact of homestays on local economic development or cultural heritage [26]. The direction of these research discussions mostly belongs to the perceived value, experience quality and satisfaction of homestay guests. However, few relevant studies have focused on the decision-making factors of how guests make homestay selection. Therefore, this study will establish a decision-making model for homestay consumers to choose homestays.

Homestays are an important alternative to traditional hotel accommodation. This type of accommodation is a comprehensive transformation of residential buildings, ecological environment and local culture [3]. Consumers can acquire aesthetic value, spiritual value, social value, historical value, symbolic value, and authenticity value from culture [27], and the beauty of goods being sold can present the historical connection, the touch of culture, or the meaning and the true value of goods. In addition, a symbiotic relationship exists between culture and tourism [28]. Therefore, by integrating the value of culture into tourism-related designs and making culture the resource for tourism product development, we can make the culture, society, and economy function together to attract more tourists and deliver a rich cultural experience. Buildings with cultural or historical significance are scattered all over Taiwan, but they gradually decline and disappear from the public’s memory. However, they can be revitalized through artistic means. Recently, Taiwan has witnessed the renovation of old department stores, the transformation of old and abandoned houses into parks and spaces with cultural significance, and the reconstruction of cultural and fashionable hotels imbued with local characteristics. Because Taiwan’s government is now paying more attention to preserving culture and displaying local characteristics, many Taiwanese buildings have been renovated into local cultural sites or characteristic homestays. Of late, Taiwan has begun to combine cultural and creative elements with the design of homestays, leading to the springing up of cultural homestays targeting specific consumer markets, respectively. Therefore, homestays can not only meet the personalized accommodation needs of guests but also play a key role in promoting economic growth and protecting traditional culture and arts [3].

### 2.2. FMCDM

To make a decision, multiple conflicting factors/criteria/attributes should be considered simultaneously to find the optimal solution. Additionally, this multi-criteria decision-making (MCDM) approach is an appropriate one [29]. MCDM provides an evaluation framework that can solve practical problems by helping decision makers to obtain effective solutions through scientific analysis methods. The popular MCDM tools that have been used in the literature include AHP, VIKOR, TOPSIS, etc. In terms of tourism research, decision support technology has been applied to help tourists choose appropriate and satisfactory hotels for their journey [30]. Many studies have focused on the multi-criteria support model of hotel selection, e.g., using the DEMATEL-ANP-MCDM model to solve independent and feedback issues and establish evaluation and relationship models [31]. In addition to using FDEMATEL and FAHP methods, the hybrid MCDM proposed in this study will also employ the MEC to truly obtain the selection attributes of consumers.

AHP is the most widely used MCDM technique [32]. This method can be used to evaluate the preference of alternatives using a hierarchy criterion [18]. Instead of providing the right decision, this strategy helps to provide the best solution to the MCDM problem. In addition to representing and quantifying problems, it also relates them to broader goals and analyzes different solutions [33]. The FAHP model incorporating fuzzy theory provides a better solution to the MCDM problems [34]. FMCDM was proposed as an effective method to solve complex problems with high uncertainty and complexity [35,36]. This mode is mostly used in models for evaluating alternatives and selected criteria by a committee of decision makers, where the applicability of alternatives and criteria and the importance weights of criteria can be evaluated with linguistic values represented by fuzzy numbers [19]. In addition, the pairwise comparison of factors by decision makers is inherently ambiguous because the linguistic expressions in pairwise judgments can also be represented by fuzzy numbers. Thus, combining fuzzy set theory and analytic hierarchy processes provides a resilient and systematic approach to prioritization, alternatives, and justification. Therefore, FMCDM can not only be close to the user’s idea, but also reduce the influence of the user’s subjectivity and improve the objectivity and practicability of the model [37].

## 3. Research Methodology

**Figure 1:**
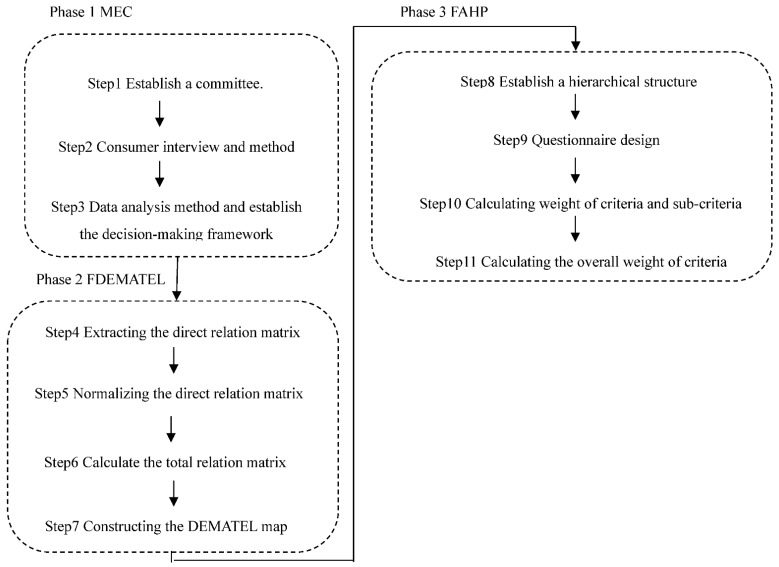
The flowchart of the research methodology.

This section illustrates the construction of a hybrid MCDM model combining with the MEC, FDEMATEL, and FAHP, which are scientific and systematic methods that can be applied effectively to obtain the purpose of this study. The proposed hybrid MCDM method is summarized in Figure 1.

### 3.1. Fuzzy Set Theory

The fuzzy set theory was proposed by Zadeh [19]. It is an effective method to represent ambiguous data. The membership function is used to denote a set, and its value lies between 0 and 1.

This study used the triangle fuzzy number (TFN) membership function to present the linguistic data. These numbers are described as $\tilde{M}=\left(l,m,u\right)$ and their membership function *μ* is defined as:

(1) $$\mu_{\tilde{M}}\left(x\right)=\{\begin{array}{c} \left(x-l\right)/\left(m-l\right),\;l\leq x\leq m\\ \left(u-x\right)/\left(u-m\right),\;m\leq x\leq u\\ 0,\;otherwise \end{array}$$

In this paper, the TFNs used to characterize the linguistic values were subjectively defined by DMs to avoid controversy and reduce sensitivity in the FDEMATEL and FAHP decision-making process. The TFNs scale can be described as: Just equal = (1, 1, 1), Equally important = (1/2, 1, 3/2), Weakly more important = (1, 3/2, 2), Strongly more important = (3/2, 2, 5/2), Very strongly more important = (2, 5/2, 3), Absolutely more important = (5/2, 3, 7/2); the reciprocal TFNs were: Just equal = (1, 1, 1), Equally important = (2/3, 1, 2), Weakly more important = (1/2, 2/3, 1), Strongly more important = (2/5, 1/2, 2/3), Very strongly more important = (1/3, 2/5, 1/2), Absolutely more important = (2/7, 1/3, 2/5) [38].

Based on the extension principle [19] and the concept of GMIR proposed by Chen and Hsieh [39], the distance between two TFNs $A_{i}=\left(l_{i},m_{i},u_{i}\right)$ and $A_{j}=\left(l_{j},m_{j},u_{j}\right)$ with distance parameter *p* = 2 can be denoted as

(2) D(Ai,Aj)={1/4[(li−lj)2+2(mi−mj)2+(ui−uj)2]}12

### 3.2. MEC

This research is based on the MEC theory. The data was collected by ladder interview method, and then using content analysis to build a hierarchical structure to determine the customers’ selection attributes, factors, and value.

Step 1: Establish a committee

This study invited experts and scholars of related industries to set up a committee to confirm the interview questions, and training of the interviewer. After, committee members will analyze the content of the interview feedback were selected.

Step 2: Consumer interview and method

Laddering [40] is the most commonly used method for MEC. Gutman [41] believes that the ladder interview method can generate and transform the product’s attributes into meaningful links. The interview method is divided into two methods: the soft ladder method and hard ladder method. When using the soft laddering method, respondents can freely answer questions without any restrictions [42]; thus, the respondents can obtain more useful information from the interviewee’s dialogue and gain a deeper understanding of the value of customers’ needs. Therefore, based on the purpose and characteristics of the present research and collecting data, the soft laddering interview method was employed.

Olson & Reynolds [15] proposed that at least 20 samples are required for ladder interviews. This research aimed at interviewing 40 guests at homestays. A pre-test interview was initially conducted with a focus group to obtain broader and diversified information through free conversation. Then, one-on-one in-depth interviews were conducted to obtain the user’s considerations through direct elicitation. Each interview lasted between 30 and 40 min. Finally, the consequences generated by attributes through the relevant analysis method were identified, and the customer’s value from these consequences was deduced [43].

Step 3: Data analysis method and establish the DM structure

Content Analysis is the MEC’s tool for interview data analysis [40]. The purpose of content analysis is to simplify the content of interviews, classify complex interview data objectively and systematically, and draw out important information for quantification cooperate with the ladder method [40,44]. This study first collected all the interview data. According to verbatim content, four professionals (two scholars and research assistants engaged in research on consumer value, MEC, and content analysis, and two professionals with more than eight years of homestay customer service experience) served as coders. After completing language stem classification and coding analysis, the link between “attribute- consequences -value” in customers’ minds was obtained, and the consumer decision attribute framework was established.

This research used reproducibility to test the reliability. Reproducibility is the degree to which more than one reviewer categorizes the classification consequences of the same content. In this study, four reviewers coded and categorized the established elements. First, the codes of the four coders were compared in pairs, and then the reliability based on the mean interrater agreement level was estimated. If the reliability exceeds the threshold of 0.85, as recommended by Kassarjian [44], it indicates high reliability. The equation is expressed as follows:

(3) Reliabliity=(n×m)/[1+(n−1)×m]

where *n* is the number of coder and *m* is the mean interrater agreement level.

### 3.3. Fuzzy DEMATEL (FDEMATEL)

DEMATEL method is a method to obtain a structural model. It can illustrate complex factors with structured cause-and-effect relationships. It also can explain the interdependence between factors in the system by causal diagram. In this study, the causal relations of accidents for construction processes can be obtain by FDEMATEL. The proposed method is suitable for presenting the relationships among the criteria and the criteria’s type of relationships. In addition, the nature of human subjective judgments can be used in linguistics terms to convert to fuzzy numbers based on fuzzy set theory. The procedures of the FDEMATEL method are explained as follows in Step 4 to Step 7 [45].

Step 4: Extract the direct relation matrix

The influential factors of this structure were defined from MEC (phase 1). After defining the influential characteristics in the research system, the experts conducted a pairwise comparison to evaluate the interactive influence degree among each pair of identified factors. Next, the linguistic measurement scale was established to compare influential characteristics pairwise.

A fuzzy direct relation matrix, ${\tilde{D}}^{e}$, was constructed by the *e*th expert; the TFNs ${\tilde{d}}_{ij}=(l_{ij},m_{ij},u_{ij}$) were used to determine the degree to which factor *I* directly affects factor *j* on ${\tilde{D}}^{e}$. The matrix can be represented by the following:

(4) $${\tilde{D}}^{e}={\left[\tilde{d}\right]}_{ij},\;i,j=1,2,\cdots ,n;e=1,2,\;\cdots ,k$$

The diagonal elements, ${\tilde{d}}_{ij}$, value was regarded as (0, 0, 0).

Based on the extension principle, the average initial direct relation matrix of the experts’ evaluation was obtained using Equation (5).

(5) $$\tilde{D}=\left({\tilde{D}}^{1}⊕{\tilde{D}}^{2}⊕\cdots ⊕{\tilde{D}}^{e}\right)/e$$

Step 5: Normalize the direct relation matrix

According to the fuzzy direct relation matrix, $\tilde{D}$, the normalized direct relation matrix $\tilde{N}$, $\tilde{N}={\left[n\right]}_{n\times n},\;i,j=1,2,\cdots ,n.$ can be obtained by using Equation (6):

(6) $${\tilde{n}}_{ij}={\tilde{d}}_{ij}/\lambda =\left(l_{ij}/\lambda ,m_{ij}/\lambda ,u_{ij}/\lambda \right)=\left(l_{ij}^{n},m_{ij}^{n},u_{ij}^{n}\right)$$

where

(7) $$\lambda =1/\underset{1\leq i\leq n}{\max}\left(\sum_{j=1}^{n}u_{ij}\right)$$

Step 6: Calculate the total relation matrix

The fuzzy total relation matrix $\tilde{T}$ was calculated using Equation (8).

(8) $${\tilde{T}}_{ij}={\left[{\tilde{t}}_{ij}\right]}_{n\times n}=\left(l_{ij}^{t},\;m_{ij}^{t},\;u_{ij}^{t}\right),\;i,j=1,2,\cdots ,n$$

where

$$\left[l_{ij}^{t}\right]=N_{l}\times {\left(I-N_{l}\right)}^{-1}\;;\;\left[m_{ij}^{t}\right]=N_{m}\times {\left(I-N_{m}\right)}^{-1};\;\left[u_{ij}^{t}\right]=N_{u}\times {\left(I-N_{u}\right)}^{-1};$$

*I* is the identity matrix.

Step 7: Construct the DEMATEL map.

In this step, the sum of each row *R_j_* (*j* = 1, 2, …, *n*) and each column *C_i_* (*i* = 1, 2, …, *n*) were calculated in the total relation matrix.

(9) $${\tilde{R}}_{j}={\left[{\tilde{r}}_{j}\right]}_{1\times n},j=1,2,\cdots ,n$$

(10) $${\tilde{C}}_{i}={\left[{\tilde{c}}_{i}\right]}_{1\times n},i=1,2,\cdots ,n$$

In the above, ${\tilde{R}}_{j}$ indicates the degree of comprehensive influence of various factors on other factors in the system, and ${\tilde{C}}_{i}$ represents the degree to which each factor is affected by other factors in the system. To finalize the procedure, all calculated (${\tilde{R}}_{j}$ + ${\tilde{C}}_{i}$) and (${\tilde{R}}_{j}$ − ${\tilde{C}}_{i}$) were defuzzified to make them suitable. The causal diagram can transform complex factor relationships into easy-to-understand graph structures to provide knowledge for problem-solving. It is built with the defuzzified (${\tilde{R}}_{j}$ + ${\tilde{C}}_{i}$) as the horizontal axis and the defuzzified (${\tilde{R}}_{j}$ − ${\tilde{C}}_{i}$) as the vertical axis. The centrality degree defuzzified (${\tilde{R}}_{j}$ + ${\tilde{C}}_{i}$) was defined as the prominence, showing the impact of the *i*th influential factor and the degree of impact.

### 3.4. FAHP

This article used the following steps of the FAHP method to help in prioritizing the criteria of evaluations: (1) establish a hierarchical structure; (2) questionnaire design; (3) calculate the weights of criteria and sub-criteria; (4) calculate the overall level weights. These steps are described in detail below:

Step 8: Establish a hierarchical structure

This paper obtained the influential attributes based on Phase 2 FDEMATEL analysis method. Through this, the AHP hierarchical framework was developed and presented.

Step 9: Questionnaire design

The AHP questionnaire in this study was designed based on the hierarchical structure described in Step 8.

Step 10: Calculate the weights of criteria and sub-criteria

Step 10.1: Establish a fuzzy pairwise comparison matrix.

The relative importance of each attribute concerning the problem’s goal was determined using the pairwise comparison matrix in which all the attributes were compared with each other, and the scores were given based on human judgment using linguistic value (shown in Section 3.1).

A pairwise comparison questionnaire is used to collect experts’ viewpoints on the relative importance of the procurement dimensions and criteria. Geometric means are more effective in integrating and conveying the opinions of many experts’ opinions compared to arithmetic means. Therefore, this study used convenience sampling to recruit food processing machinery purchasers as research participants. A total of 25 questionnaires were distributed, 20 of which were collected and considered valid, with a recovery rate of 80%. The geometric mean was used to integrate the opinions of all experts on fuzzy numbers at each hierarchy. The calculation of the fuzzy pairwise comparison matrix is as follows:

(11) A=[a˜ij]=[1a˜12⋯a˜1k1a˜121⋯a˜2k⋮⋮⋱⋮1a˜1k1a˜2k⋯1]

where ${\tilde{a}}_{ii}=1,\;{\tilde{a}}_{ij}\neq$0.

Step 10.2: Calculate the fuzzy weight values of all criteria.

The geometric mean method is used for fuzzy weights evaluation. The fuzzy geometric mean of the triangular fuzzy numbers of the *i*th evaluation dimension is calculated as follows:

(12) z˜i≅(a˜i1⊗a˜i2 ⊗…⊗ a˜ik)1k,  ∀i=1,2,…,k;

where ${\tilde{a}}_{ik}$ is the fuzzy comparison value of the *i*th criterion to the criterion *k*.

The fuzzy weight of *i*th criteria can be expressed as:

(13) $${\tilde{w}}_{i}=\left(w_{il},\;w_{im},\;w_{iu}\right)={\tilde{z}}_{1}⊗{\left({\tilde{z}}_{1}⊕{\tilde{z}}_{2}⊕\cdots ⊕{\tilde{z}}_{k}\right)}^{-1}$$

The fuzzy weight for the hierarchy of sub-criteria was constructed similarly.

Step 10.3: Defuzzify the fuzzy weight values,

The GMIR method [39] is an effective defuzzification method which is relatively simple in the calculation. To do this, ${\tilde{w}}_{i}=\left(w_{il},\;w_{im},\;w_{iu}\right)\;$ was established as the *k* triangular fuzzy weights so that the mathematical expression for *k* explicit weight values after defuzzification is as follows:

(14) $$w_{i}=\frac{w_{il}+4w_{im}+w_{iu}}{6},\;\forall i=1,2,\ldots ,k$$

Step 11: Calculate the overall weight of criteria

The *k* explicit weight values were normalized after defuzzification for obtaining the relative importance of the criteria at each hierarchy and sort the important weights: the relative importance between each layer (*L*), these normalized weights of criteria $nw_{i}{}^{L+1}$ and overall weight, $ow_{u}^{L+2}$, of *u*th sub-criteria (on the sub-criteria layer *L* + 2) under *i*th criterion with *p* sub-criteria.

(15) $$nw_{i}{}^{L+1}=\frac{w_{i}{}^{L+1}}{\sum_{i=1}^{k}w_{i}{}^{L+1}},\;\forall i=1,2,\ldots ,k$$

(16) $$ow_{u}^{L+2}=nw_{i}^{L+1}\times nw_{u}^{L+2},\;\forall u=1,2,\cdots ,p$$

## 4. Empirical Study

This study first adopted the MEC to design the framework, then the FDEMATEL was used to understand the multi-dimensional interaction, and finally, the FAHP was employed for weight analysis to confirm the perception of attributes, consequences, and values for different consumer classes.

Step 1: Establish a committee

This study invited 15 scholars and industry experts to establish a committee. The committee was tasked to discuss the main key directions of consumer interviews for homestays and the education and training of interviewers. Some personnel were also sent out to perform data analysis. Then, the DEMATEL-based questionnaire was designed for correlation. Finally, when the expert weight questionnaire using FAHP was distributed, relevant industry experts and scholars were recommended to answer the questionnaire.

Step 2: Consumer interview

Soft laddering was the main method used in this study for data collection and analysis. The interviewers directly asked respondents questions and the respondents answered without any restrictions. In this study, a total of 50 respondents who previously lived in homestay were recruited as respondents in the actual interview method by the snowball sampling method. The research interview period was from January to February 2021. Interviews were still available during this period as COVID-19 was not severe. The influential factors for selecting homestays were identified using one-on-one in-depth interviews. Each interview lasted between 30 and 45 min. Then, the possible benefits of these attributes were deduced through association and analysis. Finally, the values generated from these result benefits were obtained to explore their connections [43]. The participants who were recruited in this study were mostly females (60%, *n* = 30), aged between 36 and 50 years old (84%, *n* = 42), single (38%, *n* = 19), with a monthly disposable income of between NT$ 30,000 and NT$ 40,000 (60%, *n* = 30), and held a bachelor’s or associate degree (88%, *n* = 44).

Step 3: Data analysis and establish the DM framework

This study collected the interview data of respondents. According to the transcriptions, four professionals (two management doctors and research assistants with experience in consumer value, MEC, and content analysis, and two practitioners with over 8 years of experience in homestay services and high connections to customers) were chosen as coders. After the word stem classification and coding analysis, we explored the attribute–consequence–value hierarchy of consumers.

From the analysis of interview records, 23 attributes, 7 consequences, and 2 values were obtained (Table 1). At the attribute level, the frequent occurrences were Promotion (C1-1-1), Professional service staff (C1-2-2), Easy to book (C1-4-1), Convenient transportation (C1-4-3), and Free parking (C1-4-4). At the consequence level, Price (C1-1) was mentioned the most, followed by Convenience (C1-4) and Food and tour itinerary (C2-2). At the value level, Offer value (V1) and Leisure and cultural value (V2) were identified.

In order to ensure the reliability of the data obtained, the four professionals mentioned above participated in this research from research design to data collection, coding, and data analysis. In addition to providing practical advice, coding, and classification suggestions, they also controlled the quality of the research process to ensure the validity of the content analysis. The mean inter-rater agreement level in this study was 0.83. According to the calculation Equation (3) of reliability, the reliability was 0.91, which is greater than the reliability threshold of 0.85 [44]. This indicates that this research has good reliability. Finally, according to the results of data analysis, the basic structure of “Attribute-Consequence-Value” in the minds of customers was carried out.

Step 4: Extract the direct relation matrix

In this step, a decision-making group of experts with knowledge and experience about the problem was invited to conduct a pairwise comparison and evaluate the interactive influence degree among each pair of identified factors. A total of 15 questionnaires were distributed, and all were recovered (100% recovery rate). As for the questionnaire, the instruction was explained first, and then the respondents provided their answers. The purpose of the questionnaire was to understand the correlation and cause–effect among the criteria and factors and to identify the solution to the core problem, which will be used as a reference for the management. The linguistic measurement scale was established for the pairwise comparison among influential characteristics. Next, the fuzzy linguistic measurement scale for dealing with the vagueness of human assessments was defined, which includes: no impact = (0, 0, 0), low impact = (0.25, 0.5, 0.75), medium impact = (0.5, 0.75, 1), high impact = (0.75, 1, 1). Based on Equations (4) and (5), we obtained the direct relation matrix (Table 2).

Step 5: Normalize the direct relation matrix

According to the fuzzy direct relation matrix, the normalized direct relation matrix can be obtained using Equation (6). The normalized direct relation matrix for this study is shown in Table 3.

Step 6: Calculate the total relation matrix

The fuzzy total relation matrix was calculated using Equation (8), and the results are shown in Table 4.

Step 7: Construct the DEMATEL map

**Figure 2:**
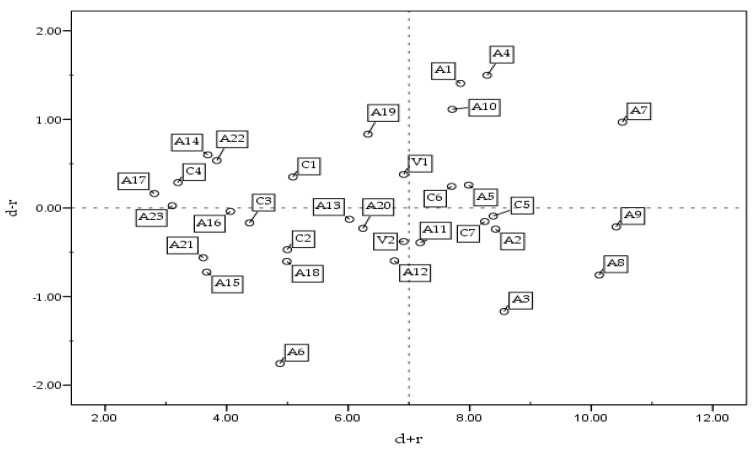
Coordinate of centrality and cause of each index.

In this step, the sum of each row $R_{j}\;\left(j=1,2,\ldots ,n\right)$ and each column $C_{i}\;\left(i=1,2,\ldots ,n\right)$ were calculated in the total relation matrix. Each column d-value and row r-value, including the direct and indirect influence between each indicator, were calculated by the defuzzify Equation (14). Accordingly, the degrees of centrality (*d* + *r*) and cause (*d* − *r*) were obtained (see Table 5). If the value of (*d* − *r*) is positive, the indicator belongs to the category of cause. If (*d* − *r*) value is negative, it belongs to the category of consequence. The category of characteristics for key success is summarized in Figure 2, and the meanings represented by each quadrant are described below:

(1) The factors falling in the first quadrant are those with core influence because they have high degrees of centrality and cause. They were listed as the objects for priority processing. The analysis results are V1, C6, A1, A4, A5, A7, A10 and A19.
(2) The factors falling in the second quadrant are those with driving forces because of their low degree of centrality and a high degree of cause. They belong to the category of cause factors, but they are also independent and have weak influence because of their low centrality. Compared with the factors in the first quadrant, they were listed as second priority objects: C1, C4, A14, A16, A17, A22, and A23.
(3) The factors falling in the third quadrant are those with interdependence due to their low degrees of centrality and cause. They belong to the category of consequence factors, but they are independent due to their low centrality. Due to their low interaction with others, it is necessary to control them separately. Compared with the factors in the first and second quadrants, they were listed as the third priority objects. These are C2, C3, A6, A15, A18, and A21.
(4) The factors falling in the fourth quadrant are influential because they have a high degree of centrality and a low degree of cause. They belong to the category of consequence. Although they are factors that should be managed, they cannot be improved in practical management but by managing factors in the first and second quadrants. Compared with the factors in other quadrants, they were listed as the last objects of management resources for processing. These are V2, C5, C7, A2, A3, A8, A9, A11, A12, A13, and A20.

This paper obtained the influential attributes according to the DEMATEL analysis method. Based on this, an AHP hierarchical framework and questionnaire were designed to determine the key factors that consumers consider when choosing a homestay. The steps in Phase 3 are Steps 8 to 11.

Step 8: Establish a hierarchical structure

The key attributes of this stage were obtained from the DEMATEL analysis. The two value criteria and ten sub-criteria are summarized as the hierarchical structure of the key criteria of the homestay selection (see Table 6).

Step 9: Questionnaire design

The content of the questionnaire was divided into three parts: the first is the description of the hierarchical structure and the content of each criterion; the second part is an example of how to fill in the AHP questionnaire to avoid invalid questionnaires or failure to meet the consistency requirements, and the third part is a comparison of the relative importance of criteria.

The questionnaire respondents included researchers or current supervisors engaged in homestay management in the academe and related industries, and their opinions were taken into consideration on the determination of weights. The survey began in February 2021; 10 academics and 10 industry experts were invited to answer the questionnaire. Some experts were unwilling to answer the questionnaire because of the complex structure of the AHP questionnaire, and it was very different from the commonly structured questionnaire. Further, some were busy during the survey administration period. Therefore, 15 valid questionnaires were collected (9 from experts in the academe and 6 from related industries).

Step 10: Calculate the weights of criteria and sub-criteria

The pairwise comparison matrix calculated the consistency index C.I. and the consistency ratio C.R. value. If the values of C.I. and C.R. are less than or equal to 0.1, it means a passed consistency [18]. The results of this paper had C.I. values and C.R. values less than 0.1, which indicates high consistency. Steps 10.1 to 10.4 were then employed to calculate the weights of criteria for each class.

Step 11: Calculate the overall weight of criteria

The integrated weight was obtained using Equations (15) and (16). The results are as follows:

**Table 6:** Integration weight of each attribute.

Values	Original Weight (C)	Attributes (A)	Original Weight	Integration Weight (C*A)	Rank
V1 Offer value	0.5500	V1.1 Promotion	0.1877	0.1032	4
		V1.2 Clean environment	0.2354	0.1295	1
		V1.3 Professional service staff	0.2056	0.1131	3
		V1.4 Facility safety	0.2095	0.1152	2
		V1.5 Easy to book	0.1618	0.0890	6
V2 Leisure and cultural value	0.4500	V2.1 Decoration and furnishings	0.2308	0.0790	7
		V2.2 Designing and creative surroundings	0.1756	0.0743	8
		V2.3 Beautiful building	0.1652	0.0895	5
		V2.4 Variety of tours and itineraries	0.1989	0.0680	11
		V2.5 Experience the local culture	0.1510	0.0687	10
		V2.6 Enjoy artistic creation	0.1526	0.0705	9

## 5. Conclusions and Discussion

### 5.1. Conclusions

This study integrated MEC, FDEMATEL, and FAHP to explore the DM attributes of homestay consumers. Based on the research results, the following conclusions are drawn:

First, MEC sequentially links product attributes to consequences of product use and individuals’ value and highlights final value goals’ connotations and formation process [41]. With soft laddering, the research completed the means-end chain theory and obtained the value hierarchy for consumers’ homestay. This could help homestay owners understand consumers’ choice behavior and psychology. The research obtains and analyzing those 23 elements, 7 were deduced; finally, two value objectives were obtained: offer value and leisure and cultural value.

Second, in the second stage of the FDEMATEL, the influential factors summarized the characteristic categories of each key success factor into four quadrants to identify the relatively influential two-quadrant factors. The factors with core influence in the first quadrant were listed as the priority objects for processing: “Promotion”, “Clean environment”, “Professional service staff”, “Facility safety”, “Easy to book” and “Variety of cities and itineraries”. The objects of the second quadrant in the second sequential processing also have five attributes: “Decoration and furnishings”, “Designing and creative”, “Beautiful building”, “Experience the local culture”, and “Enjoy artistic creation”.

Third, according to the weights of 11 influential criteria after carrying out the relative importance analysis and integration by AHP, we found that the most important decision-making factor was “Clean environment”, followed by “Facility safety”, “Professional service staff”, “Promotion”, and “Beautiful building”. The objective results obtained after three screening stages are reference criteria for both homestay operators and consumers.

### 5.2. Theoretical Contribution

In this study, by using the method of content analysis to simplify the content of interviews, classify complex interview data objectively and systematically, and draw out important information for quantification, this cooperated with the ladder method [40]. This study obtained the decision-making criteria for homestay guests to choose accommodation appropriately, and innovated the way to obtain the evaluation criteria for homestay guests. The preliminary criteria obtained from the research can be used as a theoretical reference for the diversification of homestay operations, and innovate the way to obtain the criteria for guest accommodation selection.

Secondly, the research results prove that the aesthetics of the building is a key and important factor for guests to choose accommodation, which enriches the research content of homestay selection criteria. The research results of scholars Zhao et al. [22] also mentioned that visual sensory impressions help consumers’ experience and recall. Therefore, in addition to the evaluation criteria proposed by previous scholars, we have proved from a holistic perspective that aesthetics and architecture are also key factors in the choice of homestay accommodation.

### 5.3. Managerial Implications

Based on the results, we were able to identify the factors that consumers consider when they are making decisions in homestay selection. First, based on the integrated weight results of the FAHP, this study found that most consumers value a clean environment and professional service staff when selecting homestays. This part belongs to the service quality performance of the hotel and homestay industry. For the degree of centrality (*d* + *r*) in the dimension of service quality, the clean environment criterion has the greatest impact, followed by professional service staff. In regard to the degree of cause (*d − r*), the values of two criteria, including clean environment and professional service staff, were positive; between the two, environmental cleanliness has the strongest influence. In terms of the cause–effect obtained from the comprehensive centrality and cause analysis, the clean environment was the strongest influential criterion, followed by professional service staff. Scholars Ismail et al. [25] and Qiu et al. [46] also proposed that good service quality has a positive impact on customer consumption intentions. Therefore, when aiming to improve the service quality, homestay owners must put their best effort into maintaining a clean environment and having professional service staff. According to the MEC-based consumer interview, these two attributes belonged to service quality, affecting the offer value in consumer values.

Second, the integrated weight result of the FAHP revealed that facility safety is the third most important criterion for consumers when selecting homestays. It was found that the degree of centrality (*d* + *r*) in the dimension of safety takes facility safety as the most important criterion. As for the degree of cause (*d − r*), only the value of the facility safety criterion was positive, which means that it is a cause criterion. In terms of cause–effects obtained from comprehensive centrality and cause analysis, facility safety was the main influential safety criterion. Therefore, homestay operators must focus on social safety to improve safety. This will also enrich the offer value in consumer values based on the framework of MEC.

Third, promotion and discount ranked fourth in importance when consumers select homestays. As for the degree of centrality (*d* + *r*) in the price dimension, it was found that promotion and discount have the largest influence. When it comes to the degree of cause (*d −* *r*), the value of promotion and discount was positive, which is a cause criterion. Regarding the cause–effect obtained from the comprehensive centrality and cause analysis, promotion and discount were the main influential criterion. The scholar Qiao et al. [46] believes that the pricing of homestays is important and conducts research on the pricing of homestay operators. Scholar Rasoolimanesh et al. [47] also proposed in the research on customer cognitive value that the price of homestay has a positive and significant impact on customer satisfaction. Therefore, to attract customers through competitive pricing, homestay operators must offer discounts to promote their homestay and ensure that their services are reasonably priced to enhance the offer value in consumer values.

Finally, beautiful buildings and decorations and furnishings ranked fifth and sixth, respectively. As for the degree of centrality (*d* + *r*) in the dimension of environmental experience, decoration and furnishings have the largest influence, followed by beautiful buildings. Regarding the degree of reason (*d −* *r*), the values of beautiful buildings and decoration and furnishings were both positive, suggesting that they are reasonable criteria. In terms of the cause–effect obtained from the comprehensive centrality and cause analysis, beautiful buildings and decoration and furnishings were important influential criteria. This result is connected with the research of scholars [22,27] that found that tourists have a connection with the beauty, history and culture of homestay buildings and the surrounding environment; the research of Basak [48] also pointed out that the environment and culture sustainability increases guest satisfaction. Therefore, to enhance customers’ environmental experience, the environmental design should be considered. Homestay owners must execute beautification projects and put together unique decorations and furnishings to improve the leisure and cultural value perceived by consumers.

### 5.4. Limitations and Future Suggestions

This paper has made some breakthroughs in the study of the room accommodation decision-making model, but there are still the following limitations that need to be improved. First, through a literature review and customer interviews, we identified the factors that make travelers choose homestay. However, this study may not be able to identify all types of homestay regarding travelers’ decision-making factors in choosing accommodation. Therefore, future research can combine the distinction of homestay types to collect and extract the factors of choice more widely. Second, we take guests from homestays in Taiwan as the research sample, but there are differences between homestay types and regions [2,22]. Therefore, whether the criteria used by the models are different remains to be further explored. Third, this study only calculated FAHP, and did not compare different methods. It is suggested that the comparison of different methods can be added in future research.

## Figures and Tables

**Table 1:** Frequency of each attribute, consequence, and value.

Values (V)	Freq.	Consequences (C)	Freq.	Rank	Attributes (A)	Freq.	Rank
V1 Offer value	36	C1-1 Price	37	1	C1-1-1 Promotion	38	1
					C1-1-2 Discount	30	13
					C1-1-3 Gift	16	21
		C1-2 Service quality	32	5	C1-2-1 Clean environment	33	10
					C1-2-2 Professional service staff	38	1
					C1-2-3 Free Wi-Fi	23	17
		C1-3 Safety	25	7	C1-3-1 Facility safety	34	7
					C1-3-2 Room security	15	22
					C1-3-3 Environmentally friendly	20	18
		C1-4 Convenience	36	2	C1-4-1 Easy to book	37	3
					C1-4-2 Easy payment	26	16
					C1-4-3 Convenient transportation	35	5
					C1-4-4 Free parking	35	5
V2 Leisure and cultural value	39	C2-1 Experience with the environment	33	4	C2-1-1 Decoration and furnishings	32	12
					C2-1-2 Rich with cultural elements	34	7
					C2-1-3 Designing and creative surroundings	33	10
					C2-1-4 Beautiful building	28	15
		C2-2 Food and tour itinerary	36	2	C2-2-1 Variety of delicious meals	30	13
					C2-2-2 Variety of tours and itineraries	37	3
					C2-2-3 Itinerary is easy and comfortable	34	7
		C2-3 Cultural and Creative Knowledge	31	6	C2-3-1 Full of stories	20	18
					C2-3-2 Experience the local culture	18	20
					C2-3-3 Enjoy artistic creation	15	22

**Table 2:** Direct relation matrix.

	V1	V2	C1	C2	C3	C4	C5	C6	C7
V1	(0, 0, 0)	(0.50, 0.75, 1)							
V2	(0.35, 0.60, 0.85)	(0, 0, 0)							
C1			(0, 0, 0)	(0.45, 0.70, 0.85)	(0.45, 0.65, 0.80)	(0.25, 0.40, 0.55)			
C2			(0.30, 0.50, 0.70)	(0, 0, 0)	(0.35, 0.55, 0.70)	(0.15, 0.30, 0.40)			
C3			(0.25, 0.45, 0.60)	(0.35, 0.55, 0.70)	(0, 0, 0)	(0.10, 0.20, 0.30)			
C4			(0.15, 0.35, 0.55)	(0.15, 0.35, 0.55)	(0.15, 0.35, 0.55)	(0, 0, 0)			
C5							(0, 0, 0)	(0.35, 0.55, 0.75)	(0.50, 0.75, 0.90)
C6							(0.35, 0.60, 0.85)	(0, 0, 0)	(0.30, 0.55, 0.80)
C7							(0.45, 0.70, 0.90)	(0.35, 0.55, 0.70)	(0, 0, 0)
	A1	A2	A3	A4	A5	A6	A7	A8	A9
A1	(0, 0, 0)	(0.5, 0.75, 0.9)	(0.4, 0.6, 0.75)						
A2	(0.35, 0.55, 0.7)	(0, 0, 0)	(0.3, 0.55, 0.75)						
A3	(0.3, 0.5, 0.65)	(0.3, 0.5, 0.65)	(0, 0, 0)						
A4				(0, 0, 0)	(0.55, 0.8, 0.95)	(0.2, 0.35, 0.5)			
A5				(0.35, 0.55, 0.7)	(0, 0, 0)	(0.2, 0.35, 0.45)			
A6				(0.05, 0.15, 0.25)	(0.05, 0.1, 0.15)	(0, 0, 0)			
A7							(0, 0, 0)	(0.55, 0.8, 0.95)	(0.5, 0.75, 0.95)
A8							(0.3, 0.5, 0.7)	(0, 0, 0)	(0.35, 0.6, 0.8)
A9							(0.45, 0.65, 0.8)	(0.45, 0.65, 0.8)	(0, 0, 0)
	A10	A11	A12	A13	A14	A15	A16	A17	
A10	(0, 0, 0)	(0.5, 0.75,0.9)	(0.2, 0.35, 0.5)	(0.2, 0.35, 0.5)					
A11	(0.4, 0.6, 0.7)	(0, 0, 0)	(0, 0.15, 0.3)	(0.05, 0.15, 0.25)					
A12	(0.05, 0.15, 0.25)	(0, 0.1, 0.2)	(0, 0, 0)	(0.4, 0.6, 0.75)					
A13	(0.1, 0.2, 0.3)	(0.15, 0.3, 0.45)	(0.35, 0.55, 0.7)	(0, 0, 0)					
A14					(0, 0, 0)	(0.55, 0.75, 0.8)	(0.55, 0.8, 1)	(0.2, 0.35, 0.5)	
A15					(0.05, 0.1, 0.15)	(0, 0, 0)	(0.45, 0.7, 0.85)	(0.2, 0.35, 0.5)	
A16					(0.45, 0.65, 0.8)	(0.45, 0.65, 0.8)	(0, 0, 0)	(0.25, 0.4, 0.55)	
A17					(0.3, 0.45, 0.55)	(0.25, 0.45, 0.6)	(0.2, 0.4, 0.55)	(0, 0, 0)	
	A18		A19	A20	A21		A22	A23	
A18	(0, 0, 0)		(0.05, 0.2, 0.35)	(0.15, 0.3, 0.45)					
A19	(0.25, 0.4, 0.55)		(0, 0, 0)	(0.25, 0.45, 0.65)					
A20	(0.1, 0.2, 0.3)		(0.25, 0.4, 0.55)	(0, 0, 0)					
A21					(0, 0, 0)		(0.05, 0.2, 0.35)	(0.15, 0.3, 0.45)	
A22					(0.25, 0.4, 0.55)		(0, 0, 0)	(0.25, 0.45, 0.65)	
A23					(0.1, 0.2, 0.3)		(0.25, 0.4, 0.55)	(0, 0, 0)	

**Table 3:** Normalized direct relation matrix.

	V1	V2	C1	C2	C3	C4	C5	C6	C7
V1	(0, 0, 0)	(0.50, 0.75, 1)							
V2	(0.35, 0.60, 0.85)	(0, 0, 0)							
C1			(0, 0, 0)	(0.21, 0.33, 0.40)	(0.21, 0.31, 0.38)	(0.12, 0.19, 0.26)			
C2			(0.14, 0.24, 0.33)	(0, 0, 0)	(0.17, 0.26, 0.33)	(0.07, 0.14, 0.19)			
C3			(0.12, 0.21, 0.29)	(0.17, 0.26, 0.33)	(0, 0, 0)	(0.05, 0.10, 0.14)			
C4			(0.07, 0.17, 0.26)	(0.07, 0.17, 0.26)	(0.07, 0.17, 0.26)	(0, 0, 0)			
C5							(0, 0, 0)	(0.20, 0.31, 0.43)	(0.29, 0.43, 0.51)
C6							(0.20, 0.34, 0.49)	(0, 0, 0)	(0.17, 0.31, 0.46)
C7							(0.26, 0.40, 0.51)	(0.20, 0.31, 0.40)	(0, 0, 0)
	A1	A2	A3	A4	A5	A6	A7	A8	A9
A1	(0, 0, 0)	(0.32, 0.48, 0.58)	(0.26, 0.39, 0.48)						
A2	(0.23, 0.35, 0.45)	(0, 0, 0)	(0.19, 0.35, 0.48)						
A3	(0.19, 0.32, 0.42)	(0.19, 0.32, 0.42)	(0, 0, 0)						
A4				(0, 0, 0)	(0.50, 0.73, 0.86)	(0.18, 0.32, 0.45)			
A5				(0.32, 0.5, 0.64)	(0, 0, 0)	(0.18, 0.32, 0.41)			
A6				(0.05, 0.14, 0.23)	(0.05, 0.09, 0.14)	(0, 0, 0)			
A7							(0, 0, 0)	(0.31, 0.46, 0.54)	(0.29, 0.43, 0.54)
A8							(0.17, 0.29, 0.4)	(0, 0, 0)	(0.2, 0.34, 0.46)
A9							(0.26, 0.37, 0.46)	(0.26, 0.37, 0.46)	(0, 0, 0)
	A10	A11	A12	A13	A14	A15	A16	A17	
A10	(0, 0, 0)	(0.32, 0.48, 0.58)	(0.13, 0.23, 0.32)	(0.13, 0.23, 0.32)					
A11	(0.26, 0.39, 0.45)	(0, 0, 0)	(0, 0.1, 0.19)	(0.03, 0.1, 0.16)					
A12	(0.03, 0.1, 0.16)	(0, 0.06, 0.13)	(0, 0, 0)	(0.26, 0.39, 0.48)					
A13	(0.06, 0.13, 0.19)	(0.1, 0.19, 0.29)	(0.23, 0.35, 0.45)	(0, 0, 0)					
A14					(0, 0, 0)	(0.23, 0.31, 0.33)	(0.23, 0.33, 0.42)	(0.08, 0.15, 0.21)	
A15					(0.02, 0.04, 0.06)	(0, 0, 0)	(0.19, 0.29, 0.35)	(0.08, 0.15, 0.21)	
A16					(0.19, 0.27, 0.33)	(0.19, 0.27, 0.33)	(0, 0, 0)	(0.10, 0.17, 0.23)	
A17					(0.13, 0.19, 0.23)	(0.10, 0.19, 0.25)	(0.08, 0.17, 0.23)	(0, 0, 0)	
	A18	A19	A20		A21		A22	A23	
A18	(0, 0, 0)	(0.05, 0.21, 0.37)	(0.16, 0.32, 0.47)						
A19	(0.26, 0.42, 0.58)	(0, 0, 0)	(0.26, 0.47, 0.68)						
A20	(0.11, 0.21, 0.32)	(0.26, 0.42, 0.58)	(0, 0, 0)						
A21					(0, 0, 0)		(0.18, 0.32, 0.41)	(0.12, 0.18, 0.21)	
A22					(0.32, 0.47, 0.59)		(0, 0, 0)	(0.21, 0.35, 0.47)	
A23					(0.15, 0.29, 0.41)		(0.15, 0.24, 0.29)	(0, 0, 0)	

**Table 4:** Total relation matrix.

	V1	V2	C1	C2	C3	C4	C5	C6	C7
V1	(0.21, 0.82, 50.67)	(0.61, 10.36, 60.67)							
V2	(0.42, 10.09, 50.67)	(0.21, 0.82, 50.67)							
C1			(0.09, 0.39, 10.72)	(0.29, 0.72, 20.19)	(0.29, 0.69, 10.43)	(0.16, 0.43, 10.43)			
C2			(0.19, 0.51, 10.73)	(0.09, 0.38, 10.64)	(0.23, 0.58, 10.22)	(0.11, 0.35, 10.22)			
C3			(0.17, 0.47, 10.58)	(0.22, 0.56, 10.75)	(0.08, 0.34, 10.1)	(0.09, 0.30, 10.1)			
C4			(0.10, 0.4, 10.58)	(0.11, 0.44, 10.72)	(0.11, 0.43, 0.98)	(0.03, 0.18, 0.98)			
C5							(0.16, 0.68, 40.68)	(0.31, 0.84, 40.41)	(0.38, 0.98, 40.94)
C6							(0.29, 0.87, 50.01)	(0.11, 0.54, 40.11)	(0.27, 0.86, 40.92)
C7							(0.36, 0.95, 40.93)	(0.30, 0.82, 40.31)	(0.15, 0.66, 40.51)
	A1		A2		A3		A4	A5	A6
A1	(0.18, 0.81, 20.92)		(0.46, 10.24, 40.79)		(0.39, 10.14, 60.24)				
A2	(0.32, 0.96, 20.64)		(0.16, 0.79, 40.38)		(0.31, 10.01, 50.73)				
A3	(0.29, 0.89, 20.33)		(0.31, 0.98, 30.84)		(0.14, 0.69, 50.02)				
A4							(0.21, 0.87, 40.5)	(0.62, 10.46, 50.39)	(0.33, 10.06, 40.71)
A5							(0.4, 10.05, 40.25)	(0.21, 0.85, 40.23)	(0.29, 0.92, 40.07)
A6							(0.07, 0.35, 10.83)	(0.08, 0.37, 10.94)	(0.03, 0.23, 10.63)
	A7		A8		A9	A10	A11	A12	A13
A7	(0.2, 0.82, 6)		(0.49, 10.29, 7)		(0.44, 10.22, 7)				
A8	(0.28, 0.86, 50.39)		(0.17, 0.75, 50.65)		(0.31, 0.97, 50.97)				
A9	(0.38, 1, 50.66)		(0.43, 10.13, 60.24)		(0.19, 0.81, 50.93)				
A10						(0.12, 0.5, 30.31)	(0.38, 0.9, 40.18)	(0.19, 0.67, 40.01)	(0.21, 0.69, 40.01)
A11						(0.29, 0.66, 20.85)	(0.1, 0.44, 20.93)	(0.06, 0.45, 30.03)	(0.09, 0.46, 30.03)
A12						(0.07, 0.36, 20.39)	(0.05, 0.39, 20.72)	(0.07, 0.35, 20.97)	(0.29, 0.64, 20.97)
A13						(0.12, 0.45, 20.74)	(0.14, 0.53, 30.18)	(0.26, 0.65, 3)	(0.09, 0.41, 3)
	A14		A15		A16	A17	A18	A19	A20
A14	(0.09, 0.29, 0.78)		(0.33, 0.67, 10.31)		(0.32, 0.69, 0.97)	(0.15, 0.4, 0.97)			
A15	(0.08, 0.25, 0.62)		(0.07, 0.27, 0.74)		(0.23, 0.5, 0.73)	(0.12, 0.31, 0.73)			
A16	(0.24, 0.48, 0.98)		(0.28, 0.6, 10.25)		(0.12, 0.4, 0.94)	(0.16, 0.39, 0.94)			
A17	(0.16, 0.37, 0.79)		(0.18, 0.46, 10.02)		(0.16, 0.46, 0.62)	(0.04, 0.2, 0.62)			
A18							(0.03, 0.28, 20.21)	(0.08, 0.44, 20.45)	(0.18, 0.59, 20.95)
A19							(0.34, 0.86, 30.73)	(0.12, 0.55, 30.31)	(0.33, 0.93, 40.29)
A20							(0.2, 0.63, 30.18)	(0.3, 0.75, 30.27)	(0.11, 0.52, 30.41)
	A21			A22			A23		
A21	(0.1, 0.39, 10.06)			(0.22, 0.55, 10.13)			(0.17, 0.44, 0.95)		
A22	(0.4, 0.87, 10.87)			(0.11, 0.44, 10.18)			(0.28, 0.66, 10.41)		
A23	(0.22, 0.61, 10.39)			(0.2, 0.5, 10.11)			(0.07, 0.29, 0.81)		

**Table 5:** Degree of centrality (*d* + *r*) and cause (*d − r*) values for each index.

		*d*	*r*	*d* + *r*	*d* − *r*
V1	Offer value	3.6465	3.2677	6.9141	0.3788
V2	Leisure and cultural value	3.2677	3.6465	6.9141	−0.3788
C1	Price	2.7208	2.3697	5.0905	0.3511
C2	Service quality	2.2642	2.7341	4.9983	−0.4700
C3	Safety	2.1040	2.2719	4.3759	−0.1679
C4	Convenience	1.7421	1.4553	3.1973	0.2868
C5	Experience with the environment	4.1479	4.2391	8.3871	−0.0912
C6	Food and tour itinerary	3.9729	3.7288	7.7017	0.2441
C7	Cultural and Creative Knowledge	4.0471	4.2000	8.2471	−0.1529
A1	Promotion	4.6272	3.2212	7.8484	1.4060
A2	Discount	4.0934	4.3315	8.4249	−0.2381
A3	Gift	3.6992	4.8671	8.5663	−1.1679
A4	Clean environment	4.8925	3.3946	8.2871	1.4979
A5	Professional service staff	4.1200	3.8618	7.9817	0.2582
A6	Free Wi-Fi	1.5608	3.3169	4.8776	−1.7561
A7	Facility safety	5.7404	4.7718	10.5121	0.9686
A8	Room security	4.6876	5.4437	10.1312	−0.7561
A9	Environmentally friendly	5.0993	5.3118	10.4110	−0.2125
A10	Easy to book	4.4128	3.2987	7.7114	1.1141
A11	Easy payment	3.3971	3.7876	7.1847	−0.3905
A12	Convenient transportation	3.0822	3.6781	6.7603	−0.5960
A13	Free parking	2.9467	3.0744	6.0211	−0.1277
A14	Decoration and furnishings	2.1448	1.5451	3.6900	0.5997
A15	Rich with cultural elements	1.4738	2.1976	3.6715	−0.7238
A16	Designing and creative	2.0114	2.0500	4.0614	−0.0385
A17	Beautiful building	1.4862	1.3236	2.8098	0.1627
A18	Variety of delicious meals	2.1942	2.7974	4.9916	−0.6033
A19	Variety of tours and itineraries	3.5794	2.7464	6.3258	0.8330
A20	The itinerary is easy and comfortable	3.0064	3.2362	6.2426	−0.2297
A21	Full of stories	1.5269	2.0884	3.6152	−0.5615
A22	Experience the local culture	2.1871	1.6514	3.8385	0.5358
A23	Enjoy artistic creation	1.5649	1.5392	3.1041	0.0257

## Data Availability

Data is available on request from corresponding author.
